# Compound-Protein Interaction Analysis in Condition Following Cardiac Arrest

**DOI:** 10.22086/gmj.v0i0.1380

**Published:** 2018-12-31

**Authors:** Mona Zamanian Azodi, Mostafa Rezaei Tavirani, Majid Rezaei Tavirani

**Affiliations:** ^1^Student Research Committee, Proteomics Research Center, Shahid Beheshti University of Medical Sciences, Tehran, Iran; ^2^Proteomics Research Center, Shahid Beheshti University of Medical Sciences, Tehran, Iran; ^3^Faculty of Medicine, Iran University of Medical Sciences, Tehran, Iran

**Keywords:** Heart Arrest, Protein Interaction Maps, Biomarkers, Transcriptome

## Abstract

**Background:**

Cardiac arrest (CA) and differentially expressed genes (DEGs) relative to postCA have attracted the attention of scientist to prevent damages, which threaten patients. In the present study, metabolites relevant to DEGs of post-CA condition investigated via protein-compound interaction to understand the pathological mechanisms in the human body.

**Materials and Methods:**

STITCH plug-in integrated into Cytoscape V.3.6.1 was used to detect the most significant interacting compounds relative to DEGs of pig’s brain after 5 minutes’ CA. The genes were obtained from the Gene Expression Omnibus database. The identified elements were considered for further evaluation and validation by literature survey.

**Result:**

Findings indicate that biochemical compounds including magnesium, calcium, glucose, glycerol, hydrogen, chloride, sulfate, and estradiol interact with DEGs in the two up- and down-regulated networks.

**Conclusion:**

The compounds interacting with DEGs are suitable subjects to analysis for re-regulation of the body after CA.

## Introduction

Cardiac arrest (CA) is one of the frequent life-threating situations by which the brain is the most important post affected parts [1,2]. Studise showed that CA impact on nervous system could result in long-lasting disabling [3]. Also, studies show that moderate to brain death post events are reported in 90% of heart attack survivors [4]. Therefore, neurological care is a critical issue in patients with heart attack. Understanding molecular events of CA could provide essential information of well diagnosis and also understanding post pathological outcome of the brain and vital organs of human body dysfunctions [5]. In fact, these changes start at the molecular level, which could be indicators of risk of heart disease initiation or post injuries of CA [6,7]. Meaning that identification of biomarkers related to CA can play a role in prognosis, diagnosis, and treatment approaches, and it is valuable in different states of physiological conditions. In this light, molecular modifications could be referred to risk genes, the expression difference of some genes and proteins as well as metabolite level changes and their impact in any biological processes [6,8]. For example, one study suggested that a metabolic gene, which is responsible for remodeling of phospholipids (variant near lysophosphatidylcholine acyltransferase-1) is involved in sudden heart attack [7]. However, no single molecular factor could be sufficient in prognosis and diagnosis of the risk and post outcomes of heart attack [3]. On of promising ones is the whole genome study such as array evaluation that could detect some genes with differential expression in this matter [9]. Interaction of these differentially expressed genes (DEGs) with other biochemical reagents such as metabolites could be important to evaluate them as the key important players in cellular functions [10,11]. In addition, these compounds may have an impaired level in the serum as they are linked to DEGs. In this study, the communicating compounds after identification were assigned for systematic review to evaluate and validate their possible impaired levels in the body. In other words, this network of compound-proteins could be an indicator of which elements’ level are prone to change when a specific key genes expression changes. Therefore, in this study, biochemical reagents linked to DEGs were examined to get a better knowledge of post-CA mechanisms.

## Materials and Methods

To provide a silico evaluation of protein-compound interactions, a set of DEGs after 5 minutes’ CA [3] were selected to evaluated by a bioinformatics approach.

### Data Collection

The original data was from expression array study of animal model handled by Martijn and Wiklud that was download from GEO database, GSE22165. These genes were known as 37 up-regulated (fold change ≥2) and 22 down-regulated ones in the post-CA state (fold change ≤ 0.5). In which, while Transthyretin (TTR) and GNRHR had the highest amount of expression change among all.

### Protein-Protein Interaction Analysis

Considering centrality properties, GNRHR and PRL displayed the highest priorities in post-cardiac condition after 5 minutes [3].

In this analysis, two up- and down-regulated groups were separately examined for compound interaction analysis by the use of STITCH Plug-in situated into Cytoscape V. 3.6.1. In a way that, the list of gene names were queried in STITCH platform, and two criteria were designated for this aim [12]. The STITCH platform is the part of STRING database that offers platforms for network construction via four different sources including STRING, STITCH, DISEASES, and PubMed. Edge score and number of maximum additional interactions are assigned as 0.4 and 10, respectively. However, not all DEGs were retrieved in our network query due to the unrecognition of some via STITCH Database. The research continued by exploring the identified compounds against literature to improve the validity of the results. The search strategy for this review was by exploring google scholar and Medline from 1975 to 2018. The data of compounds that showed associations with CA was gathered and discussed in more details in the study. The conflicting studies about the linkage of the recognized biochemical reagents were also acknowledged and mentioned in the investigation.

### Statistical Analysis

Fold change more than 2 and less than 0.5, and a P value≤0.05 were considered to determine significant gene expression change and relative compounds.

## Result

The introduced significant DEGs [3] were considered to construct a protein-compound network of up- and down-regulated genes. The two networks were handled by STITCH compound query, a Cytoscape 3.6.1.

**Figure 1:**
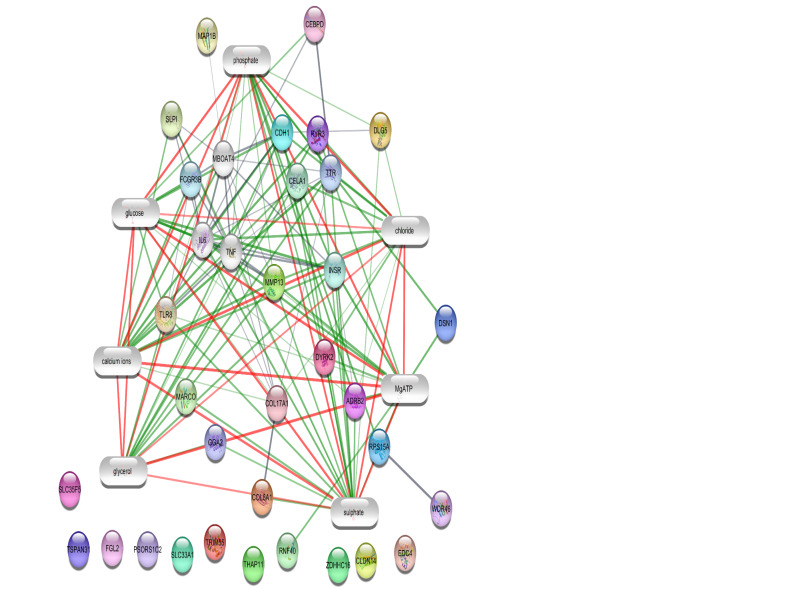
Network view of up-regulated genes of CA and their corresponding metabolites. Nodes with grey color are the added ten interactors. The round shaped nodes are proteins, and the elliptical are compounds. In this network, ten genes remained as induvial by not entering the main network. Number of nodes and interactions were 42 and 133, respectively. Links in green, red, and grey colors are the connections between protein-compound, compound-compound, and protein-protein, respectively.

**Figure 2:**
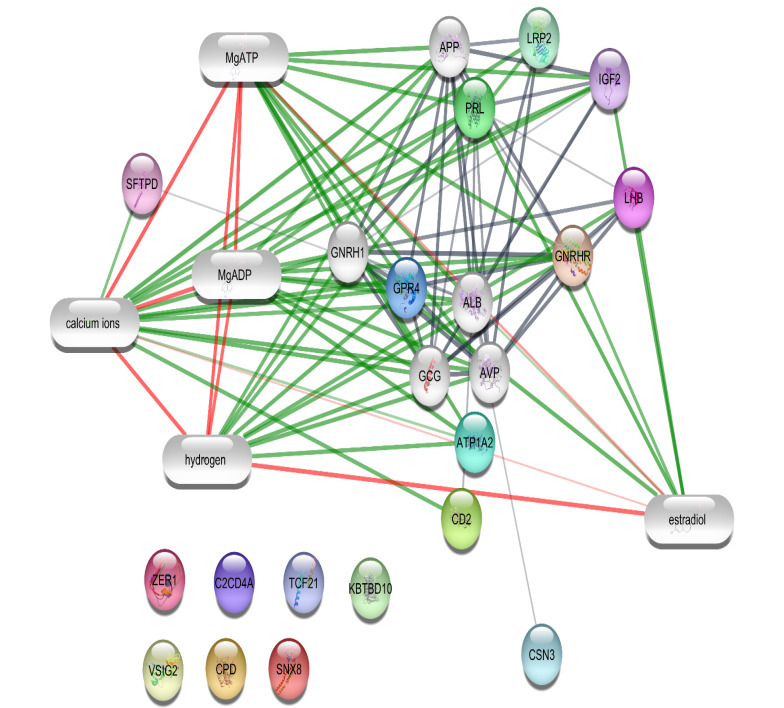
Network view of down-regulated genes of CA and their corresponding metabolites. Nodes with grey color are the added ten interactors. The round shaped nodes are proteins, and the elliptical shapes are compounds. In this network, seven genes remained as induvial by not entering the main network. Number of nodes and interactions were 27 and 98, respectively. Links in green, red, and grey colors are the connections between protein-compound, compound-compound, and protein-protein, respectively.

Plug-in (Figures-1 and 2). Ten compounds; Mg-ATP, sulfate, glucose, glycerol, calcium ion, chloride, phosphate, Mg-ADP, hydrogen, and estradiol were included in the networks, which were extracted from the networks and presented in Table-1. To validate the finding, level change of the introduced compounds in CA samples were searched and considered (Table-1).

**Table1:** The List of Ten Compound Names and Their Relations

**Name**	**URN**	**DRN**	**NIG**		**NIC**		**Interactor score**		**Level**	**References**
**Mg-ATP**			11	10^*^	6	4	7.45	4.58^*^	↓	20, 29
**Sulfate**		-	16		6		11.66		-	-
**Glucose**		-	9		6		5.23		↑	17, 18
**Glycerol**		-	10		5		6.54		-	-
**Calcium**			10	13^*^	6	3	7.53	6.81^*^	↓	27, 30
**Chloride**		-	10		6		6.4		-	-
**Phosphate**		-	11		6		6.7		-	-
**Mg-ADP**	-		10		3		4.55		↓	20, 29
**Hydrogen**	-		9		3		4.53		-	-
**Estradiol**	-		5		3		3.75		↓	31

**URN:**up-regulated network; **DRN:**down-regulated network; **NIG:**number of interacting genes; NIC: number of interacting compounds ↑: increase; ↓: decrease *quantities are depended to DRN

## Discussion

Biochemical level changes in CA condition are studied in different investigations [13,14]. The knowledge of this fact could be beneficial in the outcome of patient care and consequently valuable in reducing post injuries in one of the most sensitive organs in human body namely brain [15].

In our research, ten biochemical compounds were identified that regulation of their relevant genes were affected in CA patients. The following discussed documents confirm the findings and reveal the importance of regulation of the introduced metabolites in the survivors.

Gene expression profiling provided some essential understanding at this level that leads to deciphering pathological mechanisms.

Some studies (GSE70107 and GSE22165) on animal model showed that in the heart attack condition, vast genes come across fundamental expression changes that studying them could be beneficial for this goal. In the present study, the significant DEGs are screened to determine associated biochemical reagents. In this way, it may be possible to add some more information about the molecular pathology at this condition. Through this study, the DEGs from the research (GSE22165) and our previous bioinformatics approach [3] were chosen for further analysis in terms of interacting biochemical.

A network of interacting gene-compound for each type of regulation has been constructed as it is shown in Figures-1 and 2. The queries were done by adding ten elements to the main set of up- and down-regulated genes. Our results indicate that neighbor genes and its compounds are retrieved by the queries. In the up-regulated network, 70% of the added nodes including glucose, calcium ions, glycerol, sulfate, chloride, Mg-ATP are from compounds while in the down-regulated network, the number of added genes and compounds (hydrogen, estradiol, calcium ions, Mg-ATP and Mg-ADP) are equal. Calcium ions and Mg-ATP are two compounds that are common between both up- and down-regulated networks.

In this study, we focus on the roles and the possible previous reports of the retrieved compounds. All the compounds show high amounts of interactions in the two networks. In particular, sulfate shows the highest degree of all and is in connection with other compounds and genes. Evaluating these compounds by literature offers more evidence. First of all, the alternation of many metabolites following CA has been suggested by many studies including free fatty acids, glucose, and catecholamine [16].

Glucose is one of the most reported compounds, which is a highly interacted element of the up-regulated network. Glucose impaired levels and its association with a heart attack are suggested by many studies. In other words, hyperglycemia has been detected in most patients with CA [17,18]. Glucose increment is correlated to up-regulation of the insulin receptor. It may be affected by insulin resistance or insulin deficiency. Mg as another interacting compound in both networks of up- and down-regulation has been supported by many studies that have preventive and therapeutic effects in heart attack [19,20]. For instance, Woods and Fletcher reported that magnesium sulfate prescription in 2316 patients with acute myocardial infarction led to a reduction in mortality rate from ischemic heart disease and all origins to 21% and 16%, respectively [21]. It is well-known that magnesium plays crucial role in many biological reactions in the body. Mg deficiency is associated with cardiac arrhythmias. It is an important regulatory element of K+-ion channels that are essential in the electric conductive activity of the heart [22]. As it is shown in Table-1 levels of Mg-ATP and Mg-ADP are decreased after CA. According to the most studies, prescription of 17β-estradiol has a neuroprotective effect after CA [23-25]. As it is shown in Figure 2 and Table-1 estradiol level is decreased after CA; therefore, it can be concluded that neuroprotective activity is suppressed and neuron system is exposed to damage after CA. The low concentration of calcium ion has a relationship with a heart attack. Calcium increment levels promote ATP production in mitochondria [26,27]. Investigations indicate that calcium ion has an essential role in cardiac function. Regulation of calcium concentration during reperfusion after ischemia has an important impact on the proper function of the heart [28]. Calcium is related to both networks and is involved via highly connections style. It seems that calcium level regulation is depended to various genes due to its precise role in the control of heart function. Since immediate treatment after CA can compensate several lesions and prevent many damages, the introduced panel of compounds can be considered as metabolite candidates, which should be balanced in patients with CA.

## Conclusion

We show that the determined compounds, which are related to DEGs may be balanced after heart attack in survivors immediately.

## Conflict of Interest

The authors declare no conflict of interest.
